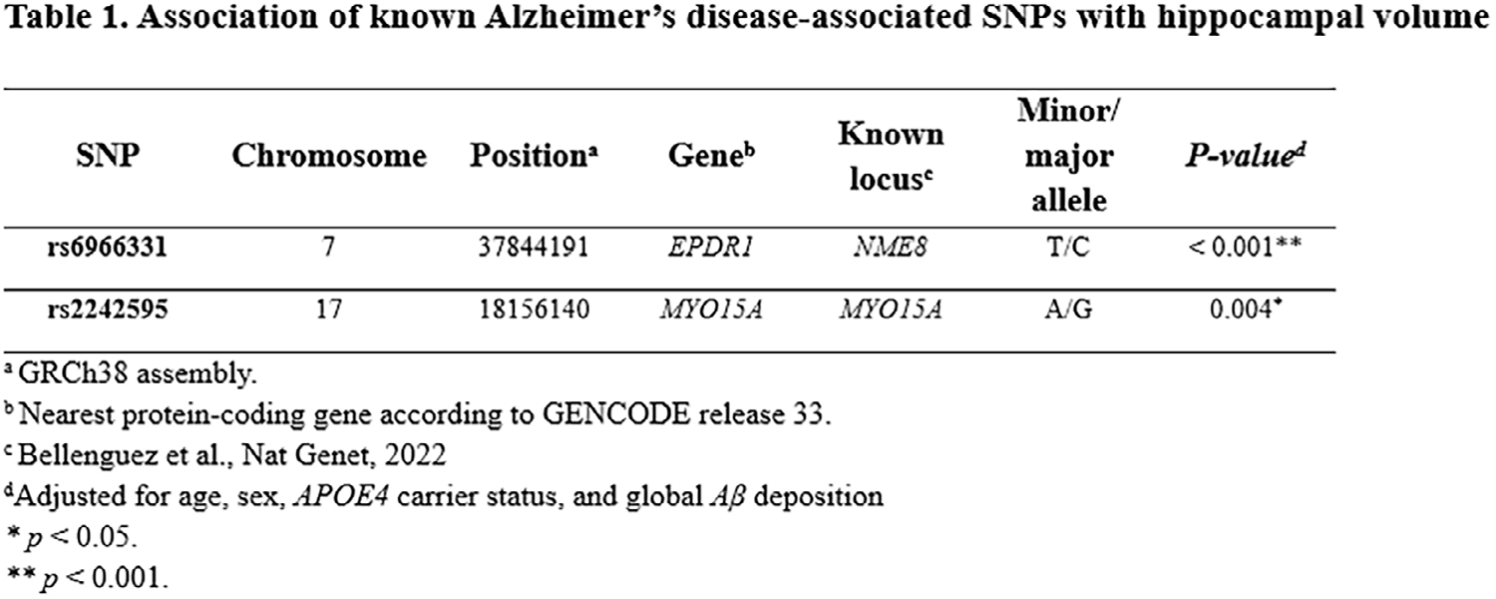# Association between known Alzheimer’s disease risk genetic variants and hippocampal atrophy along the Alzheimer’s disease continuum in a Korean cohort

**DOI:** 10.1002/alz.092461

**Published:** 2025-01-03

**Authors:** Hyejin Ahn, Min Soo Byun, Dahyun Yi, Gijung Jung, Yen‐Ning Huang, Shannon L. Risacher, Anthony J. Griswold, Margaret A Pericak‐Vance, Yu Kyeong Kim, Yun‐Sang Lee, Chul‐Ho Sohn, Koung Mi Kang, Jun‐Young Lee, Andrew J. Saykin, Kwangsik Nho, Dong Young Lee

**Affiliations:** ^1^ Interdisciplinary program of cognitive science, Seoul National University College of Humanities, Seoul Korea, Republic of (South); ^2^ Department of Psychiatry, Seoul National University College of Medicine, Seoul Korea, Republic of (South); ^3^ Seoul National University Dementia Research Center, Seoul Korea, Republic of (South); ^4^ Department of Neuropsychiatry, Seoul National University Hospital, Seoul Korea, Republic of (South); ^5^ Institute of Human Behavioral Medicine, Medical Research Center, Seoul National University, Seoul Korea, Republic of (South); ^6^ Center for Neuroimaging, Department of Radiology and Imaging Sciences, Indiana University School of Medicine, Indianapolis, IN USA; ^7^ Indiana Alzheimer’s Disease Research Center, Indiana University School of Medicine, Indianapolis, IN USA; ^8^ John P. Hussman Institute for Human Genomics, University of Miami Miller School of Medicine, Miami, FL, USA, Miami, FL USA; ^9^ 1501 NW 10th Avenue, Miami, FL USA; ^10^ Department of Nuclear Medicine, SMG‐SNU Boramae Medical Center, Seoul Korea, Republic of (South); ^11^ Department of Nuclear Medicine, Seoul National University College of Medicine, Seoul Korea, Republic of (South); ^12^ Department of Radiology, Seoul National University Hospital, Seoul Korea, Republic of (South); ^13^ Department of Neuropsychiatry, SMG‐SNU Boramae Medical Center, Seoul Korea, Republic of (South); ^14^ Department of Medical and Molecular Genetics, Indiana University School of Medicine, Indianapolis, IN USA; ^15^ Center for Computational Biology and Bioinformatics, Indiana University School of Medicine, Indianapolis, IN USA

## Abstract

**Background:**

Large‐scale genome‐wide association studies (GWAS) of Alzheimer’s disease (AD) from European ancestry identified many genetic variants associated with clinical diagnosis of AD dementia. However, it remains unclear whether these AD‐related variants are associated with AD biomarkers, particularly hippocampal atrophy, a well‐known neurodegeneration biomarker of AD in a Korean population. In this study, we investigated the association between known AD risk single nucleotide polymorphisms (SNPs) and hippocampal atrophy along the AD continuum in older Korean adults.

**Method:**

A total of 487 participants (258 cognitively normal olde adults [CN], 144 mild cognitive impairment [MCI], 85 AD dementia) from the Korean Brain Aging Study for the Early Diagnosis and Prediction of Alzheimer’s disease (KBASE) were included for analysis. All participants underwent ^11^C‐PiB‐PET/MRI. Hippocampal volume adjusted for intracranial volume (HVa) was obtained from 3D T1‐weighted MRI scans using FreeSurfer and used as a neurodegeneration marker of AD. Global beta‐amyloid (Aβ) deposition was calculated from PiB uptake in the global cortical region‐of‐interest using SPM12. From the genetic evidence gathered by the AD Sequencing Project (ADSP), which consists of 76 SNPs associated with AD, we selected 38 SNPs with a minor allele frequency (MAF) greater than 1% from the genotyping data imputed using the TOPMed imputation server in the KBASE cohort.

**Result:**

Among 38 known AD‐related SNPs, three SNPs (rs6966331 in *EPDR1*, rs2242595 in *MYO15A*, and rs17125924 in *FERMT2*) were associated with HVa in an initial exploratory analysis (*p*<0.05). In a subsequent confirmatory analysis, the associations of rs6966331 in *EPDR1* and rs2242595 in *MYO15A* with HVa remained significant after controlling for age, sex, and *APOE4* carrier status, as well as global Aβ deposition (*p*<0.001 and *p* = 0.009 for rs6966331 and rs2242595, respectively) (Table 1).

**Conclusion:**

Our study identified associations of rs6966331 in *EPDR1* and rs2242595 in *MYO15A* with hippocampal volume in Korean older adults, and these associations were independent of cerebral Aβ deposition and *APOE4* carrier status. These findings suggest that these AD‐related loci may contribute to the development of AD dementia *via* Aβ‐independent neurodegeneration.